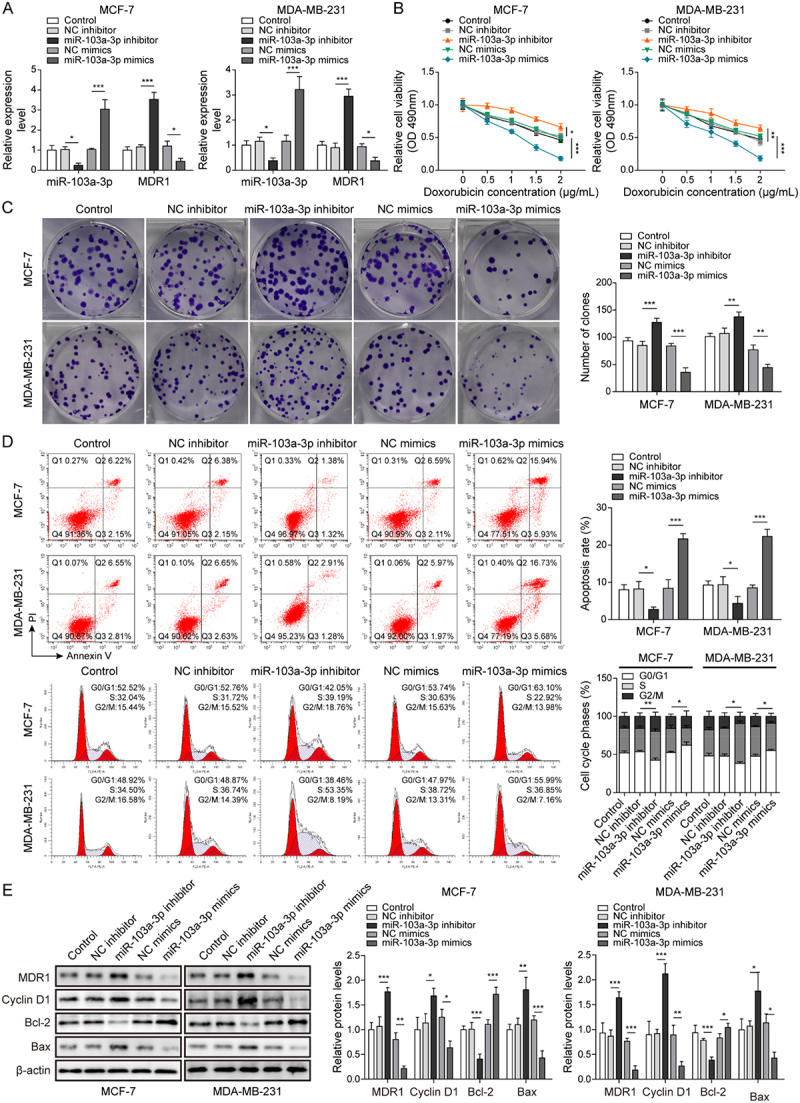# Correction

**DOI:** 10.1080/15592294.2024.2345564

**Published:** 2024-04-25

**Article title**: METTL3-mediated m6A modification of lnc KCNQ1OT1 promotes doxorubicin resistance in breast cancer by regulating miR-103a-3p/MDR1 axis

**Authors**: Zhiyang Zhou, Yukun Cao, Yuan Yang, Shouman Wang, and Feiyu Chen

**Journal**: *Epigenetics*

**Bibliometrics**: Volume 18, Number 01, pages 1-17

**DOI**: https://doi.org/10.1080/15592294.2023.2217033

It has been noted by the authors that Figure 1A, Figure 2B, Figure 3B, Figure 4B, Figure 5F, Supplementary Figure 1B, and Supplementary Figure 2B have some incorrect data. The corrected figures have been placed below. This correction has not changed the description, interpretation, or the original conclusions of the article. The authors apologize for any inconvenience caused.

**Figure 1:**
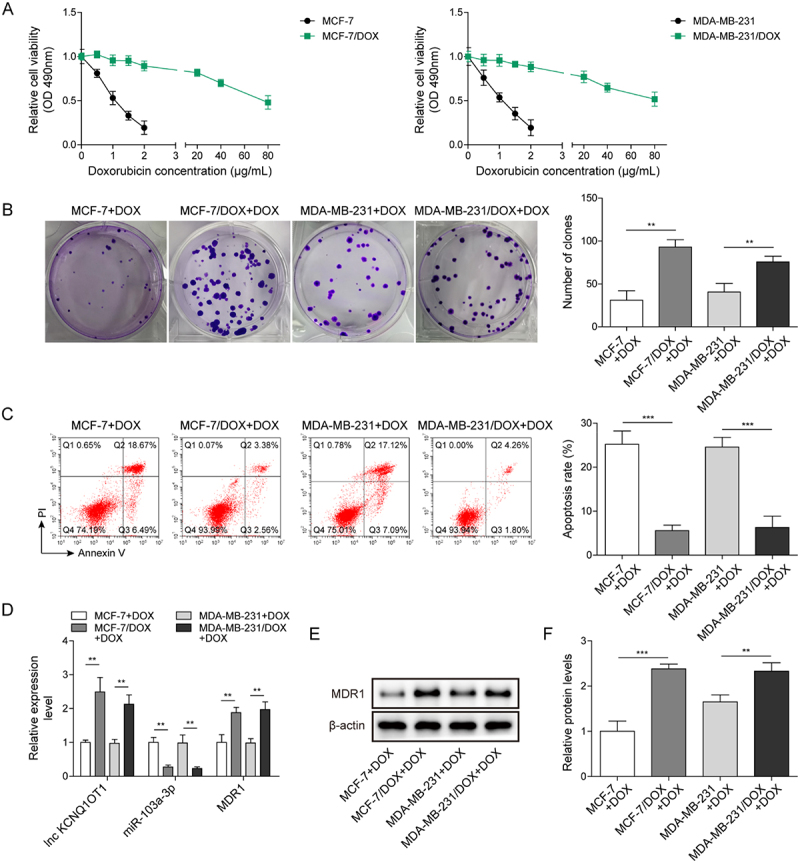

**Figure 2:**
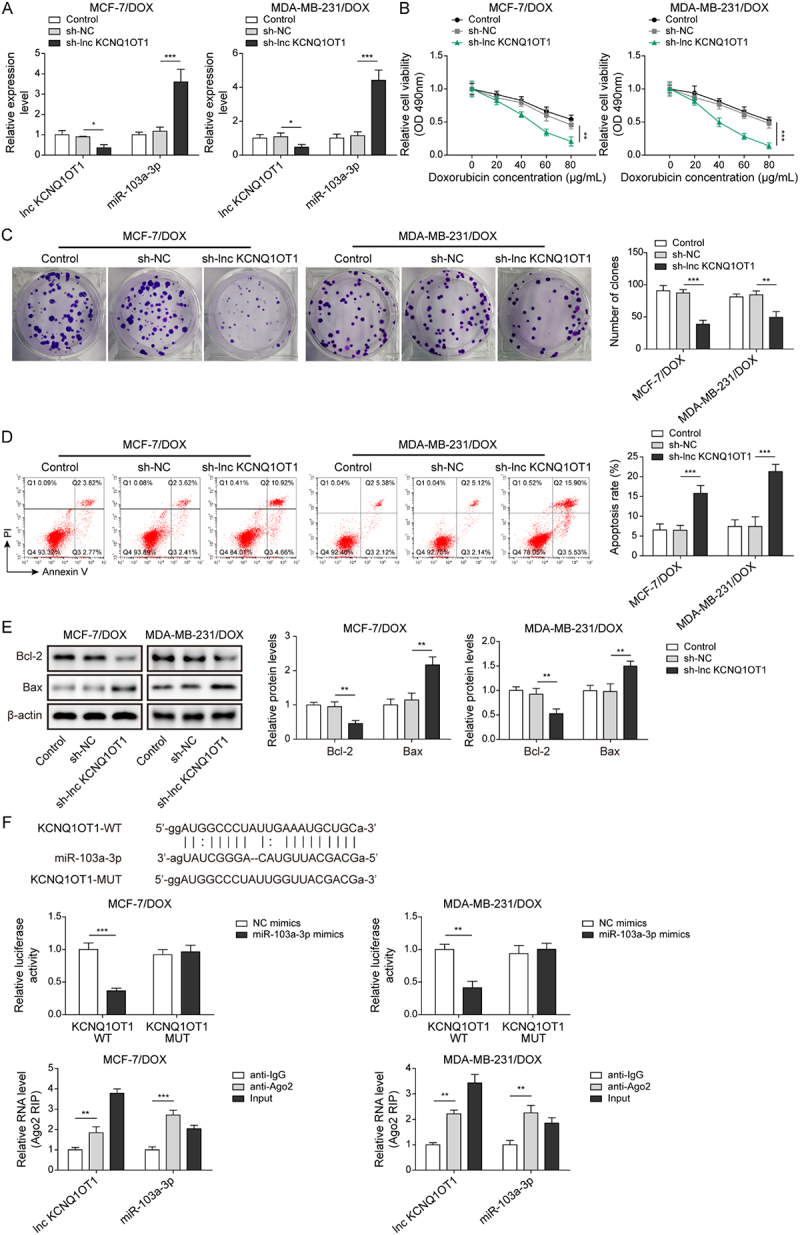

**Figure 3-1:**
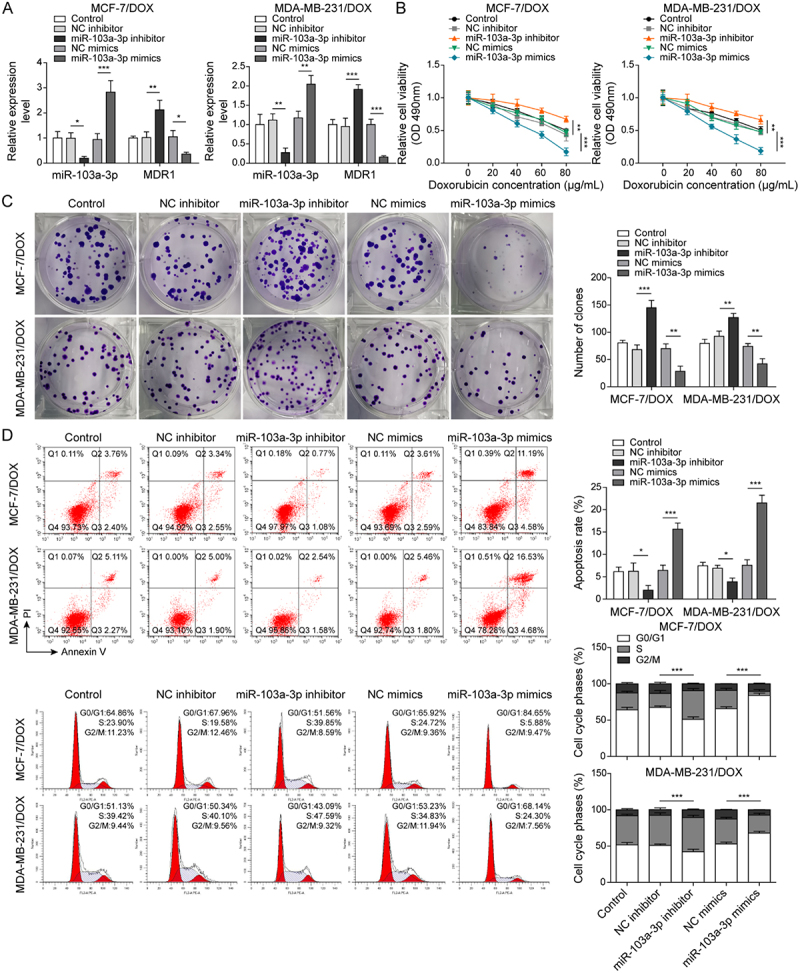

**Figure 4-1:**
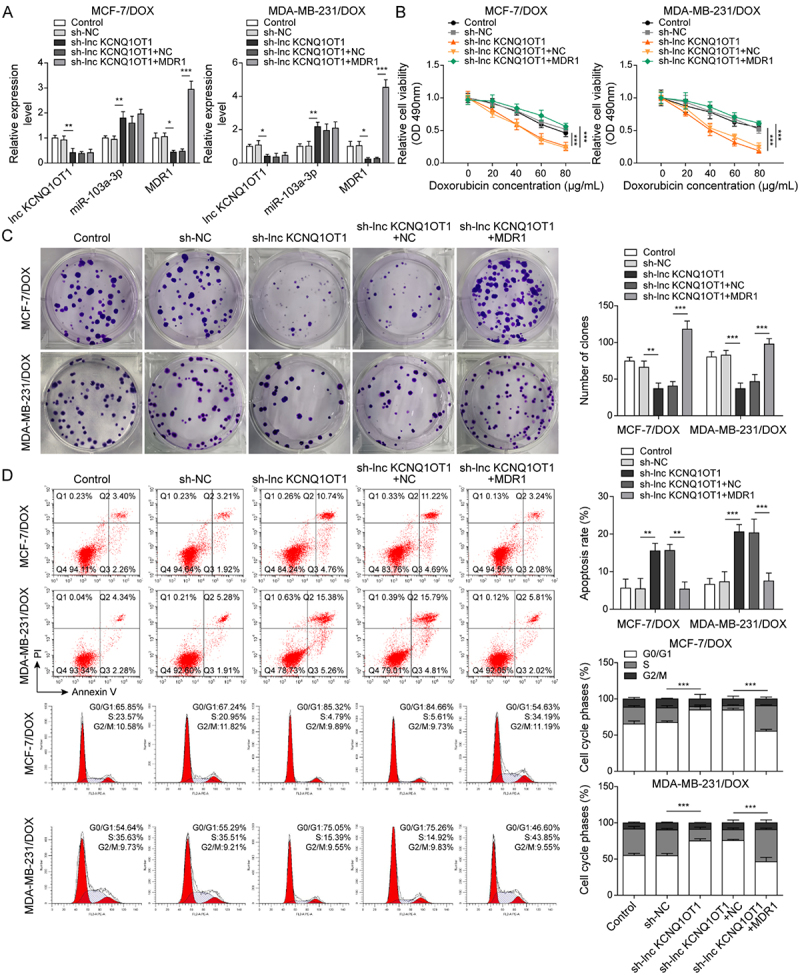

**Figure 5-1:**
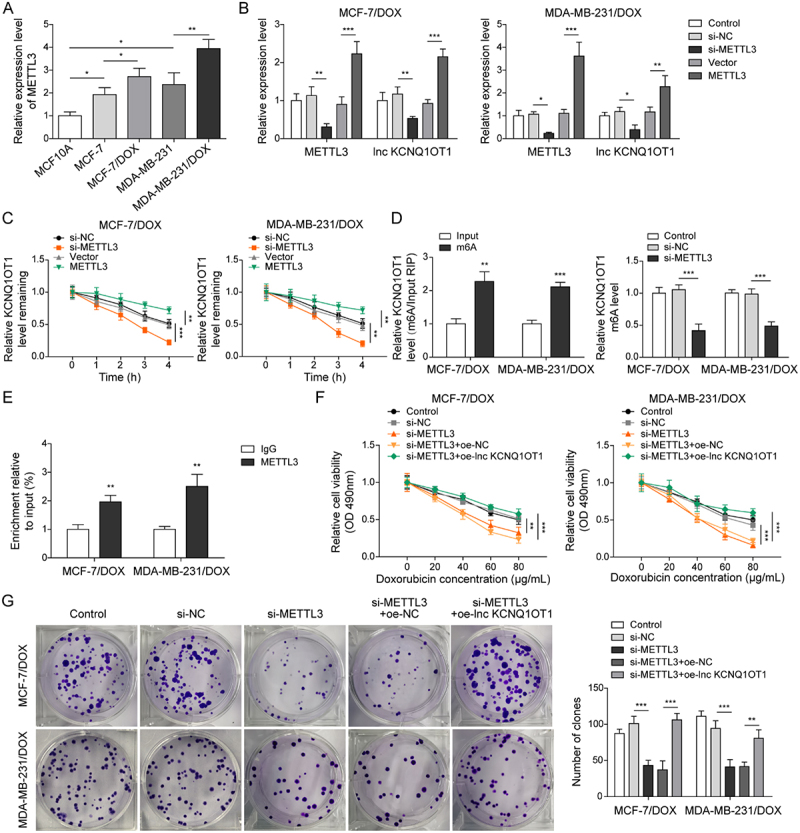

**Supplementary Figure 1:**
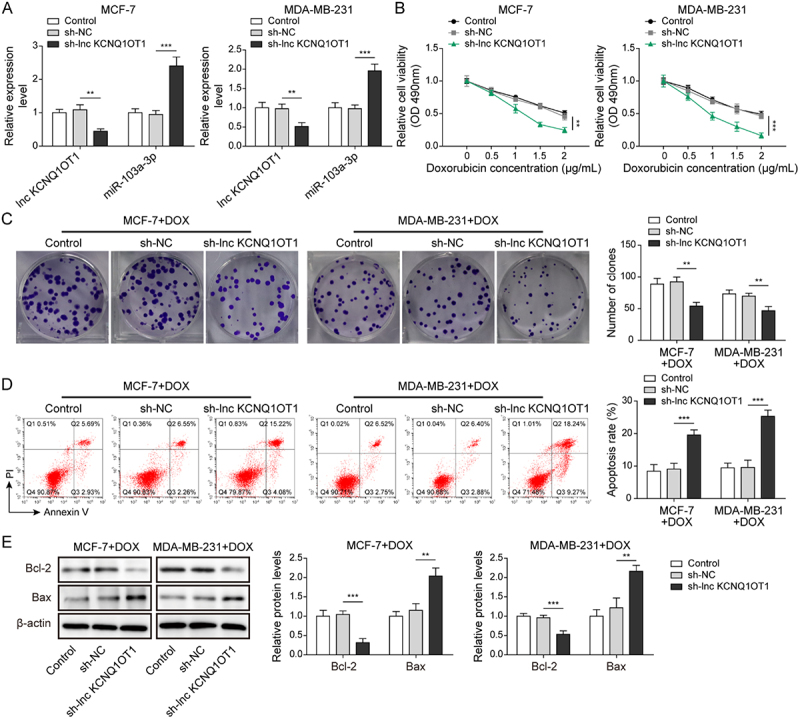

**Supplementary Figure 2:**